# Do different foot types affect the 6-min walk test capacity of younths with Charcot-Marie-Tooth neuropathy ?

**DOI:** 10.1186/s12887-022-03338-7

**Published:** 2022-05-13

**Authors:** Cyntia Rogean de Jesus Alves de Baptista, Beatriz Garcia, Juliana Cardoso, Adriana Nascimento Elias, Beatriz Parra Buzzetti, Ana Claudia Mattiello-Sverzut

**Affiliations:** 1 Laboratory of Musculoskeletal Structure and Function, Department of Health Science of Ribeirão Preto Medical School (FMRP-USP), Ribeirão Preto, São Paulo, Brazil; 2 Rehabilitation and Functional Performance Program Ribeirão Preto Medical School (FMRP-USP), Ribeirão Preto, São Paulo, Brazil; 3 Paulista University and Moura Lacerda University, Ribeirão Preto, Brazil

**Keywords:** Gait capacity, Childhood, Foot posture, Hereditary motor sensory neuropathy, Timed function tests

## Abstract

**Background:**

The present study aimed to assess the gait capacity of youths with Charcot Marie Tooth disease (CMT), considering the different foot postures as a grouping variable.

**Methods:**

The total distance, the predicted distance, and gait velocity obtained during the six-minute walking test (6MWT) were compared between participants with and without CMT. In addition, part of the CMT group completed a 12-month follow-up. The study evaluated 63 participants (CMT group = 31; Non-CMT group = 32) aged 6 to 18, both sexes. Data included anthropometric measures, foot posture index (FPI), the distance (D6), percentage of predicted distance (%D6), and walking velocity(V) in 6MWT.

**Results:**

The D6% presented no significant difference between the types of feet in CMT or Non-CMT (p < 0.05, Kruskal Wallis test). CMT presented reduced values of D6, %D6, and V when compared to Non-CMT.

**Conclusions:**

These findings indicate that gait performance was decreased in youths with CMT in comparison to non-CMT. Contrary to what was expected, the cavus foot type did not show lower gait capacity than the flatfoot, suggesting that the types of feet alone did not determine differences in gait capacity within the CMT group

**Supplementary Information:**

The online version contains supplementary material available at 10.1186/s12887-022-03338-7.

## Introduction

The foot deformities have a high incidence in Charcot Marie Tooth disease (CMT), with more than 70% of cases presenting cavus [1, 2]. Despite the varied genotypes, CMT subtypes have typical signs and symptoms, such as distal muscle weakness, sensory and myotatic ankle reflexes changes, as well as hand deformities [3, 4] From a functional perspective, children with CMT have decreased walking ability and physical activity [5], which in due course impair participation in daily activities and gradually decrease quality of life [6, 7].

For children with CMT, changes in gait and kinematics of gait are relatively well documented [8–10], and a deterioration in performance in the 6-min walk test (6MWT) associated with musculoskeletal changes in the lower limbs in a longitudinal study [5]. However, it is unclear whether the different foot postures or foot deformities affect the gait capacity. Unlike adults affected by the disease, children with CMT still do not have defined foot posture patterns nor defined deformities since aspects of the biomechanical musculoskeletal development coexist with anomalies peculiar to the current disease itself.

Inferring about gait capacity of different foot types from timed tests, such as 6MWT, has the advantages of having a low cost, speed, and broad clinical applicability compared to other instrumentalized evaluations. The 6MWT mainly evaluates ambulation ability and aerobic resistance at a submaximal level [11] and was adapted from the running test for healthy people lasting 12 min. The 6MWT has good reliability and sensitivity in adults with CMT [12, 13] and other neurological diseases [14–16], establishing relationships with global measures of functional impairment [12, 17], with postural balance and the ability to develop physical activities [13]. Reference values of the pediatric population about the 6MWT are available in the literature for specific age groups [18–22]. The 6MWT speed in healthy children increases with height [22–24]. Children with CMT present low walking velocity [10], and a study developed by Kennedy et al. (2017) [5] describes that the performance in 6MWT was inversely proportional to age; that is, the greater the age, the shorter distance traveled in 6MWT and attributed this finding to the disease progression along with somatic growth. Therefore, detecting changes resulting from pathological conditions in children with CMT through further investigations about 6MWT data, considering their feet types or deformities, can guide the selection of therapeutic procedures that prevent the decrease in gait capacity before adulthood. Our hypothesis considers that children with CMT and cavus feet should have a reduced distance in 6MWT than those with flat or normal feet.

In this context, the present study aimed to assess the gait capacity with the 6MWT, considering the different foot types as a grouping variable of children and adolescents with CMT. Comparisons between participants with CMT considered the physical variables obtained in 6MWT according to foot types (normal, cavus, and pronated). Moreover, comparisons tested differences between CMT participants and their unaffected peers. Finally, the study also carried out a 12-month follow-up on participants with CMT.

## Materials and methods

The Ethical Committee approved this study of the Clinics Hospital of Ribeirão Preto Medical School—University of São Paulo (process 14,904/2014). The parents and the patients/participants provided their written informed consent to participate in this study.

The participants diagnosed with CMT (*n* = 31) from the Neurogenetics Clinic—(ANGE) of HCFMRP-USP were recruited and evaluated between August 2016 and February 2020. Another age-matched non-affected participant (*n* = 32) came from a private school in the city of Ribeirão Preto. Intergroup analyzes involved 63 participants, 31 from the CMT group and 32 from the Non-CMT group. The follow-up analysis included 12 participants with CMT who underwent assessment 1 (EV1) and a second assessment (EV2) after 12 months (maximum of 14 months and a minimum of 10 months).

Inclusion criteria for all participants were: age between 6 and 18 years, independent walking, without previous foot and ankle surgery. After acceptance, anthropometric data, Foot Posture Index (FPI)_ [25, 26] and the 6MWT, according to a modified version of American Thoracic Society (ATS) [11] were collected at the HCFMRP – USP Rehabilitation Center.

The 6MWT occurred on a 20-m course with a flat surface. Verbal reinforcement was provided every minute to walk as fast as possible without running. For monitoring and safety, blood pressure, heart rate, and oxygen saturation were recorded in resting conditions immediately after the 6-min test ended, 10 min after the test (recovery period). The obtained variables were total distance covered (D6), the percentage of the predicted distance (D6%) obtained using the Geiger equations (for males D6% = 196.72 + (39.81 Age)—(1.36 Age^2^) + (132.28 Height; for females D6% = 188.61 + (51.50 Age)—(1.86 Age^2^) + (86.10 Height), and the average velocity (V).

The Foot Posture Index (FPI) was applied to classify the foot types [25, 26]. Photographs of the feet were obtained in an orthostatic position using a Samsung 301 camera positioned 20 cm from the participant. The evaluated items were the talar head position, curves above and below the lateral malleolus, calcaneal inversion, and eversion, talonavicular prominence, congruence of the medial longitudinal arch, and forefoot abduction and adduction over the hindfoot. The total score results from the sum of the items previously mentioned and can vary from -12 to 12, with 0 to 5 (normal foot), from 6 to 9 (pronated foot), from 10 to 12 (very pronated foot), from -1 to—4 (supinated foot) and from -5 to -12 (very supinated foot) [26].

The initial exploratory analysis used the SPSS program (version 17.0) to identify the behavior of the variables of interest: distance covered (D6), percentage of the predicted distance (D6%), average velocity (V), and FPI. The Shapiro Wilk test verified whether the variables had a normal distribution. Subsequently, an intergroup evaluation compared the 6MWT of participants with CMT with their healthy peers using the Mann–Whitney U test considering the D6, D6% based on a predictive equation (Geiger et al. 2007) and V. To assess the differences in the follow-up of the CMT group, the Student t-test paired compared the D6% data obtained at two different moments (EV1 vs. EV2). The Kruskal Wallis test tested D6 differences according to the types of feet, respecting the inserted group (CMT or Non-CMT).

## Results

### Sample characterization

Anthropometric characteristics of the CMT and Non-CMT groups are in Table 1, and, according to the results of the T-Student test, there was no significant difference between the groups in terms of age, weight, and height. As for the type of foot, there was a predominance of supinated feet in CMT, while neutral feet predominated in Non-CMT (Table 1).

**Table 1:** Anthropometric data (sex, age, weight, height, and foot type) of the CMT and Non-CMT groups

	CMT(*n* = 31)			Non-CMT (*n* = 32)	
Boys	18			17	
Girls	13			15	
		mean	SD	Mean	SD
Age (years)		12.19	2.83	12.12	2.72
Weight (kg)		50.62	14.66	44.65	12.65
Height (m)		1.53	0.13	1.53	0.14
Genotype		CMT1A = 70.97% (*n* = 22) CMT1B = 6,45%, (*n* = 2) CMTX1 = 12.90% (*n* = 4) CMT2N = 3,.23% (*n* = 1) CMT2K = 3.23% (*n* = 1) Under investigation = 3 .23% (*n* = 1)		Not applicable	
Foot Type					
Normal		35.48% (*n* = 11)		87.50% (*n *= 28)	
Pronated		9.68 (*n *= 3) %		6.25% (*n* = 2)	
Supinated		54.84% (*n* = 17)		6,25% (*n* = 2)	

### Intra-group analysis: comparisons of 6MWT values according to the foot type.

The foot types found in the CMT group did not show significant differences in the D6% (p < 0.05, Kruskal Wallis test) (Table 2). Similarly, the Non-CMT group also did not show significant differences in D6% according to the foot types (Table 2).

**Table 2:** D6% values of the CMT and Non-CMT group according to the foot types

	**CMT**			**Non CMT**		
D6%	Normal (*n* = 11)	Pronated (*n* = 3)	Supinated (*n* = 17)	Normal (*n* = 28)	Pronated (*n* = 2)	Supinated (*n* = 2)
Mean (sd)	75.54 (10.41)	70.98 (8.63)	67.11 (9.64)	85.93 (11.51)	80.89 (3.84)	79.75 (8.72)
Maximum	91.41	77.35	86.88	121.33	83.61	85.92
Minimum	59.88	61.16	53.19	64.85	78.17	73.58

### Inter-group analysis: comparisons of 6MWT values between CMT and Non-CMT group

The comparison of the CMT with the Non-CMT group, regardless of the foot type, showed significant differences in D6, D6%. and V (Mann–Whitney U test. p < 0.05). The CMT group showed lower values than Non-CMT, suggesting reduced gait capacity of the CMT group (Table 3).

**Table 3:** Comparison of the total distance covered (D6). the percentage of the total distance covered to the predicted (D6%). and average gait speed (V) between the groups

	CMT (*n* = 31)			Non-CMT (*n* = 32)		
	D6 (m)	D6% (%)	V (m/s)	D6 (m)	D6% (%)	V (m/s)
Minimum	360.00	53.19	1.00	400.00	64.85	1.11
Maximum	621.00	91.41	2.00	791.00	121.33	2.20
Mean (SE)	469.23* (11.90)	70.64* (1.88)	1.3* (0.04)	566.28 (13.5)	85.22 (1.95)	1.56 (0.05)
SD	66.27	10.51	0.25	76.73	11.05	0.27

Legend: *SE*  Standard error, *SD*  Standard deviation, *p < 0.05 when compared with Non-CMT group

### CMT group follow-up

In 12 months, re-evaluation comprised twelve participants in the CMT group, and there was a significant difference in weight and height between the evaluations (EV1—weight = 46.9 kg, SD = 16.47; height = 1.48 m, SD = 0.14 and EV2—weight = 51.34 kg, SD = 16.42; height = 1.54 m, SD = 0,14). However, there was no significant difference in physical variables between EV1 and EV2 (EV1 mean values: D6 = 446 m, SD = 61.46; D6% 68.73, SD 9.1, and V = 1.2 m/s, SD = 0,18; EV2 (mean values: D6 = 464 m, SD = 54.68; D6% 69.91, SD8.2. and V = 1.3 m/s, SD = 0.13).

## Discussion

The purpose of this study was to evaluate whether the different types of feet of youths with CMT would differ in terms of gait capacity measured through the 6MWT. The hypothesis was that cavus foot, highly prevalent in CMT [1] could have reduced performance compared to other types of feet has not been confirmed. However, the 6MWT of participants with CMT was significantly decreased when compared to their healthy peers. Furthermore, the 12-month follow-up showed no significant changes in the CMT group, both in the distance covered in absolute values (D6) and in the percentage of the predicted distance (D6%) extracted from the 6MWT.

Regarding the foot type, the term "normal" was applied in the present study and respected the original designation used in the FPI, in which scores from 0 to 5 denote a structurally aligned foot. In the healthy children population, the prevalence of pronated feet varies from 0.6 to 77.9%, with a tendency to decline overgrowth [27, 28]. The occurrence of the foot types in our study is in line with the literature because 87.50% of our Non-CMT group had normal feet [28, 29]. Participants with CMT showed a predominance of supinated feet (54.84%), as it also corroborates the findings related to the disease [1, 30, 31]. Comparing the foot types with the percentages of the distances predicted in the 6MWT did not reveal significant differences. Such results suggest that the reduced gait capacity in terms of distance and mean velocity is present in children with CMT, regardless of their foot type.

Because it is a complex disease, neuromotor impairments, muscle weakness, and distal somatosensory losses may add up to produce balance and gait deficits [32]. Among the various associations tested by Estilow et al. (2019) [32], the deformities of the feet of children with CMT had a weak association with a balance subscale (BOT-2. Bruinicks Oseretsky test), and as for the 6MWT, participants with cavus feet had the worst results. The foot type classification using the FPI occurs in a static condition; it may not reflect the foot's behavior in dynamic conditions, according to a study of radiological data and kinematics [33]. Another factor to be considered is that our study participants, unlike adults with CMT, are still developing their deformities. For this reason, there seems to be a limited impact on gait capacity, or the participants who manage to perform 6MWT tend to be those with mild and moderate impairment, similarly to what occurred in a study with adult CMT [13]. Besides, children with CMT appear to develop various compensatory walking strategies, such as increased hip and knee flexion in the swing phase [8, 10]; with that, they manage to maintain performance in 6MWT.

The intra-group analysis (EV1 vs. EV2) did not detect changes in the physical variables of the 6MWT, despite the significant changes that occurred in somatic growth. Thus, the interpretation of these findings pointed to worsening in the locomotor performance for CMT after 12 months since the 6MWT remained lower than predicted values. For Kennedy et al. (2017) [5], the decrease in 6MWT values in 12 months was evident. It is noteworthy that phenotypic variability allows varying levels of gait impairment [4], and it is not easy to select studies that are comparable with each other regarding the frequency of CMT subtypes. Age was not a clustering factor in our study, while Kennedy et al. (2017) [5] subdivided their participants into two age groups (older and challenging than 12 years). There were decreases in 6MWT only in participants older than 12 years [5]. Our follow-up included a restricted number of participants (*n *= 12) with a majority between 6 and 12 years of age (*n* = 8), a reason to explain our attenuated results.

Considering that distal deformities in childhood and adolescence are still ongoing and the classification of foot types is limited to static conditions, the results of the present study prompt us to find other factors that determine a subnormal locomotor performance of the youth with CMT. Such factors could involve signs and symptoms present in CMT since childhood, such as pain resulting from foot deformities, muscle weakness, fatigue in the lower limbs, or even a reduction in confidence and fear of falls.

As limitations of our study, it is worth noting the inexpressive number of supinated feet in the non-CMT group (*n* = 2) when compared to the CMT group, which restricted the comparison using 6MWT data. Another limitation was the age group, which can be considered broad-based on the sample size. It was impossible to subdivide participants by adopting two age groups (minors and over 12 years old) to advocate distinct development in the feet of children and adolescents [34] or to allow comparisons with the available literature[5]. The use of %D6 helped to overcome this last limitation.

## Conclusion

Analyzed in isolation, the types of feet classified by the FPI did not determine differences in gait capacity between participants with CMT, assessed by the 6MWT. However, gait capacity was reduced in participants with CMT to their unaffected peers and predicted values. The stagnation of 6MWT values in the 12-month follow-up of participants with CMT should be considered a negative result in the light of somatic and motor development.

## Supplementary Information

**Additional file 1.** 

## Data Availability

We declare that all the raw data supporting the conclusions of this article are included in its supplementary information files.
